# The Use of Wearable Sensors for Preventing, Assessing, and Informing Recovery from Sport-Related Musculoskeletal Injuries: A Systematic Scoping Review

**DOI:** 10.3390/s22093225

**Published:** 2022-04-22

**Authors:** Ezio Preatoni, Elena Bergamini, Silvia Fantozzi, Lucie I. Giraud, Amaranta S. Orejel Bustos, Giuseppe Vannozzi, Valentina Camomilla

**Affiliations:** 1 Department for Health, University of Bath, Bath BA2 7AY, UK; e.preatoni@bath.ac.uk (E.P.); lucie@teganet.eu (L.I.G.); 2 Centre for Health and Injury and Illness Prevention in Sport, University of Bath, Bath BA2 7AY, UK; 3 Department of Movement, Human and Health Sciences, University of Rome “Foro Italico”, Piazza L. de Bosis 6, 00135 Rome, Italy; elena.bergamini@uniroma4.it (E.B.); a.orejelbustos@studenti.uniroma4.it (A.S.O.B.); valentina.camomilla@uniroma4.it (V.C.); 4 Interuniversity Centre of Bioengineering of the Human Neuromusculoskeletal System (BOHNES), University of Rome “Foro Italico”, Piazza L. de Bosis 6, 00135 Rome, Italy; 5 Department of Electrical, Electronic, and Information Engineering “Guglielmo Marconi”, University of Bologna, Viale Risorgimento 2, 40136 Bologna, Italy; silvia.fantozzi@unibo.it; 6 Health Sciences and Technologies—Interdepartmental Centre for Industrial Research, University of Bologna, Viale Risorgimento 2, 40136 Bologna, Italy

**Keywords:** biomechanics, exercise, athlete, movement analysis, prevention, injury mechanisms, rehabilitation, accelerometer, inertial sensors, force transducers

## Abstract

Wearable technologies are often indicated as tools that can enable the in-field collection of quantitative biomechanical data, unobtrusively, for extended periods of time, and with few spatial limitations. Despite many claims about their potential for impact in the area of injury prevention and management, there seems to be little attention to grounding this potential in biomechanical research linking quantities from wearables to musculoskeletal injuries, and to assessing the readiness of these biomechanical approaches for being implemented in real practice. We performed a systematic scoping review to characterise and critically analyse the state of the art of research using wearable technologies to study musculoskeletal injuries in sport from a biomechanical perspective. A total of 4952 articles were retrieved from the Web of Science, Scopus, and PubMed databases; 165 were included. Multiple study features—such as research design, scope, experimental settings, and applied context—were summarised and assessed. We also proposed an injury-research readiness classification tool to gauge the maturity of biomechanical approaches using wearables. Five main conclusions emerged from this review, which we used as a springboard to propose guidelines and good practices for future research and dissemination in the field.

## 1. Introduction

Sport and physical exercise are increasingly promoted as part of a healthy lifestyle [1]. However, increased participation in physical activity and sport specialisation [2] may raise the risk of injury, especially in younger individuals [3], for whom sport-related accidents are a leading cause of medical attention and emergency department attendance [4,5,6]. The burden of sports injuries and their potential impact on quality of life and societal costs call for research and effective interventions in all of the areas associated with sports injury: prevention [3], assessment, and recovery [3,7].

Several strategies for the prevention of [8,9] and recovery from [7] injury have been proposed, alongside models of injury causation [10,11,12], classifications of injury factors (e.g., intrinsic vs. extrinsic; modifiable vs. not modifiable) [13], and reviews of the different approaches that could be adopted to study injury mechanisms [12].

From a mechanical perspective, musculoskeletal injuries occur when the load applied to a tissue goes beyond the maximum amount of mechanical energy that bodily elements can accept without compromising their structure and function [14,15]. Several theories have been developed to explain the occurrence of injuries [11]; all recognise the complexity and multifactorial nature of injury causation, and distinguish between acute and overuse injuries. In acute events, the inciting energy exceeds the maximum tolerated by the tissues involved. In overuse injuries [16], the repetitive nature of the demands sustained by the body may reduce its tolerance levels to a point where normally acceptable loads can cause micro- or macro-failures. Repetitive submaximal microtraumas can lead to a cascade of alterations to structural proprieties, function, and behaviour, which eventually establish a vicious loop of degeneration, adaptation, and pain [17,18,19].

Biomechanical approaches can contribute substantially to the study of sports injuries and their prevention. For example, they can describe injury mechanisms and characterise inciting events, or can assess the effects of interventions on movement behaviours and the ability to withstand mechanical loads. Biomechanical tools also have the potential to help monitor compliance, quality, and progress of movement performance when an injury-prevention or return-to-activity programme is implemented [3,14,20]. In vivo, in vitro, and in silico methods have been used to quantify the biomechanical demands generated by sports actions [15], together with the responses of bodily tissues that are subjected to those loads. However, measuring mechanical quantities in real-world settings—either directly or indirectly—is extremely difficult, and sometimes impossible, because of ethical considerations and lack of adequate technology or sports regulations. In most cases, the assessment is confined to controlled lab conditions [21].

The ongoing development and increased use of wearable technologies, either in isolation or as part of integrated approaches, offers an opportunity to collect quantitative data “in the field”, less obtrusively, for extended periods of time, and with fewer spatial limitations than conventional motion-capture technologies [22,23,24,25,26,27,28,29,30,31,32,33,34,35,36,37,38,39,40,41,42,43,44,45,46]. New-generation sensors are small, portable, minimally obtrusive, affordable, and easy to use; they may provide real-time feedback [36], as well as enable prospective studies on large cohorts [47].

Several review articles have assessed the use of such devices in different areas of sport science, when applied to the characterisation of sport-specific movements [22,24,34,40,42,45]; performance analysis and enhancement [23,26,27,28,44,46]; the evaluation of tactical variables [33,48]; the monitoring of load and inertial forces [29,30,37]; the trends and projections in the consumer sports sector [49]; the description of specific disciplines such as running [35,50], sprinting [27], swimming [28,51], combat sports [52], or Paralympic sports [26]; and the assessment of rehabilitative interventions [32,39]. Injury risk mitigation has been addressed within specific sport and injury domains, such as that of running-related injuries [35,36,39], head impacts [31], anterior cruciate ligament reconstruction [53,54,55], and dynamic stability in return to sport [41]. However, it appears that no work has systematically investigated the current state of the art on the role of wearable sensors in the different stages of injury assessment, including the characterisation of injury mechanisms, and the provision of information to support preventive or rehabilitative interventions.

The use of wearable sensors in movement science and sport is widespread; however, their application is still in an “exploratory phase” [22], and is not free from pitfalls [23,25,38], suggesting that both the technology and the associated methods still require further development and careful analysis [43]. Indeed, some of the features that make wearables attractive can also limit their applied impact. For example, the possibility to collect data continuously in an uncontrolled environment can generate the problem of handling large datasets affected by measurement noise, which generates the need for adequate awareness of data quality (e.g., prior validation, care in calibration procedures) and for the use of appropriate processing methods, such as machine learning, for key performance indicator estimates [56,57], or advanced data science techniques for data synthesis or prediction [58]. Moreover, there is still little evidence on the causal relationships between specific biomechanical assessments, quantities derived thereof, and injury or injury risks [36,59,60], which can result in many studies being descriptive of the potential of new technologies rather than fully exploiting that potential to unveil the relationships between biomechanics (e.g., movement technique) and injury-related features (e.g., inciting factors, recovery status).

We present a systematic scoping review [61] on the use of wearable technologies for the study of musculoskeletal sports injuries, with the aim to discuss (a) the current literature contributing to the identification and description of the biomechanical factors and mechanisms associated with injury, as well as the biomechanical evaluation of preventive or rehabilitative interventions; (b) the strength of evidence brought about by the experimental approaches used by those studies; (c) the time setting in relation to injury, primary scope, and features of the experiments; and (d) the characteristics of the technologies and types of measures used. In analysing these items, we highlight strengths and weaknesses of the current state of the art, identify existing guidelines and common pitfalls, and discuss current trends and future directions. With a view to outlining the maturity of the research in the area and guiding initiatives aiming to fill existing gaps of knowledge, we also propose (e) a simple tool for the classification of biomechanical methods employing wearable technologies in the musculoskeletal injury area. This framework, which we called the *Injury-research Readiness Level* (IrRL), is inspired by the *technology readiness level* [62] and *system readiness level* [63] frameworks; it aims to capture the maturity, functionality, and environmental readiness of biomechanical approaches to be effectively deployed in the field.

## 2. Materials and Methods

### 2.1. Protocol, Search Strategy, and Inclusion Criteria

The PRISMA-ScR [64] framework guided the systematic scoping review, which was registered in PROSPERO (registration no. CRD42021140485) on 26 March 2021. Three main stages were followed to identify and filter studies: (1) definition of databases for article retrieval, search terms, and selection rules; (2) screening based on article titles and abstracts; and (3) final selection based on full-text examination (Section 3.1).

The literature search and management were conducted using *Covidence* [65]. The *Web of Science*, *Scopus*, and *PubMed* electronic databases were browsed up to 8 January 2021. Only peer-reviewed journal articles published in English were considered for inclusion, with no a priori removal based on study design or publication date. The search strategy was based on the PICOS tool [66], and search terms were chosen to scan the literature and identify studies that used wearable technology to perform biomechanical analyses of sports activities and contribute to the area of musculoskeletal injuries (see Table S1 in the Supplementary Materials for a full description of the Boolean search terms used).

Although no restriction was imposed on the types of technology used, we primarily focused our search terms on wearable magneto-inertial sensors and force or pressure transducers. Articles reporting exclusively on activity monitoring from global navigation satellite systems, injury surveillance without biomechanical measurements, metabolic behaviours (e.g., energy expenditure), or neuromuscular activity from electromyography were excluded. All levels of sport participation were accepted, but studies on clinical populations or not including human participants (e.g., with anthropomorphic test dummies or simulators) were excluded. Injuries affecting the nervous system, such as mild traumatic brain injuries, were not considered.

Items in Stage 1 were discussed and agreed by the whole group of authors. The reference list generated by the initial search, with titles and abstracts, was stored in *Covidence* and screened independently by two researchers (S.F. and G.V.), who identified additional relevant studies, eliminated duplicate sources, and then performed Stage 2 selection, reporting no disagreement. Review papers were initially considered to better analyse the wider context of the state of the art, but no review paper was included for more detailed analysis past Stage 2, because they did not explicitly report original findings. For each article reaching Stage 3, the description of the relationship between biomechanical quantities and musculoskeletal injury was evaluated by two independent reviewers (E.B. and V.C.). Only articles directly investigating or making explicit reference to existing evidence of the relationship between the biomechanical quantities presented and musculoskeletal injury mechanisms or risk factors were included. Because of the large number of articles passing through to Stage 3, the final full-text examination was shared equally between authors, and weekly consensus meetings were held to discuss and resolve any uncertainty about the eligibility of a study. Summary statistics concerning journal and year of publication were reported, both in absolute terms (number of articles or articles per year), and normalised to the overall number of manuscripts published in the broader field of biomechanics of sport injuries. To estimate the normalising factors, we carried out a MeSH term search in PubMed, using the following query: ((sport[MeSH Terms]) AND (injury[MeSH Terms])) AND (biomechanics[MeSH Terms])), and then exporting values between 1968 (i.e., the first year available) and 2020.

### 2.2. Study Classification and Assessment

We assessed and reported on multiple feature domains of the studies selected (Table S2 in the Supplementary Materials): (1) strength of evidence, time setting, and primary scope; (2) study characterisation in terms of experimental conditions and setting, injury of interest, type and location, population tested, type of sport and motor task, and level of sporting participation; and (3) characteristics of the technologies and types of wearable device and measures used. We also defined and assessed (4) the injury-research readiness level (IrRL).

#### 2.2.1. Strength of Evidence, Time Setting, and Scope

Two independent researchers (L.G. and E.P.) assessed the strength of evidence of each article and assigned them to three main categories in decreasing order of strength, based on the experimental design used: experimental, i.e., meeting the requirements of randomised controlled trials; quasi-experimental, i.e., including a manipulation of the experimental conditions under which participants perform sport, but lacking random assignment or group comparison; and observational, i.e., without assessing the effects of an intervention, and only describing participant behaviour [67]. A separate class was used for studies looking exclusively at the validation of new equipment or methods. The opportunity to use a finer classification of observational studies (e.g., cross-sectional, case–control, cohort, case series) and include the assessment of the risk of bias of individual studies [67,68] was considered. However, given the scoping nature of the review, and in light of the large extent of literature covered by the topic, and of the variety of outcome measures and experimental approaches, we deemed this impractical and unnecessary. Indeed, our review aimed at characterising the current state of the art in a specific area of sports biomechanics and, thus, did not require formalisation of a specific experimental question, nor assessment of the quality of the studies attempting to answer such a question [61].

A *pre*/*at*/*post* classification was used to express the chronological relationship between the experimental data collected and the injuries studied. Studies were classified as *pre* if data were collected without any injury occurring, or without a direct attempt to establish a causal relationship between biomechanics and injury. These studies typically relied on findings from the existing literature to construct their rationale and hypothesis, and then discussed injury prevention implications starting from those assumptions. Articles were allocated to the *at* category if the scope of the experiment was to identify and characterise injury factors or mechanisms, and therefore attempted to capture or track injury occurrences (e.g., cohort studies with biomechanical screening and in-field injury events recorded during a sports season). It should be noted that these studies did not necessarily use wearables to identify, count, or depict injury events. Lastly, studies were recorded as *post* if data collection was performed after the injurious event, during the recovery phase, and aimed to advise rehabilitation and reduce the likelihood of injury reoccurrence. For clarity, articles on participants who had already returned to full activity (e.g., comparisons between healthy individuals and people with a history of a specific musculoskeletal problem) were classified as *pre*, because they were not centred on the recovery process that goes from injury occurrence (or medical intervention, if relevant) to being able to return to full activity.

The classification according to the primary scope was carried out based on the following categories: studies analysing sport-related injury mechanisms; studies assessing sport-related injury factors or injury risks; studies attempting to establish injury threshold criteria from a mechanical perspective; studies characterising protective devices; and studies focusing on post-injury monitoring or return-to-play assessment. Validation studies were classified according to the primary aim for which the method or tool tested had been devised, as stated by the authors. Articles addressing multiple issues were reported in more than one category, where appropriate.

#### 2.2.2. Study Characterisation

To describe experimental conditions, we extracted information about the settings in which data were collected, i.e., laboratory- vs. field-based. Specifically, studies were labelled as field-based if data were acquired during training, simulated training, or competition in a sport-specific setting. Conversely, investigations carried out within a laboratory, or in the field but using instrumentation typically adopted in the laboratory—such as obtrusive motion-capture setups or force platforms—were labelled as laboratory-based.

The injuries addressed by the works reviewed were classified as caused by overuse or acute events [16], and listed by the tissue affected (soft vs. skeletal) and body location. Furthermore, we annotated the type of sport and motor task addressed by each study and the level of sport participation of the people partaking in the tests (i.e., sedentary, recreationally active, trained/developmental, highly trained/national level, elite/international, world-class, or not specified/insufficient data to be classified) [69].

#### 2.2.3. Technical Features and Validation

The following features were taken into account when discussing wearable devices: type of measurement system (e.g., accelerometer, gyroscope, magnetometer, force or pressure transducers); brand; physical and technical characteristics (e.g., mass, dimension, full-scale capacity, resolution, buffer, voltage sensitivity, sampling frequency); number of units used to collect data; locations (on body segments or equipment); and fixing technique (e.g., pocket in a vest/belt/tape, bi-adhesive on the skin, tape, elastic strap, rigid frame). We also reported on the quantities measured via wearable sensors being essential (i.e., primary outcome), complementary (i.e., part of the analysis but not fundamental), or marginal (i.e., used for other experimental needs, e.g., equipment synchronisation) for answering the research questions, as well as the information reported about previous validations of the measurement used.

#### 2.2.4. Injury-Research Readiness Level

Building on the system readiness level (SRL, [63]) framework, we modelled the Injury-research Readiness Level (IrRL) to capture the maturity, functionality, and environmental readiness of the studies aiming to contribute to preventing, assessing, or recovering from sport-related musculoskeletal injuries. According to the SRL model, technology and system development follow similar maturation paths, whereby technology is inserted into a system and interacts with it via a proposed architecture. Knowing about the system components and their integration [70] is essential, and this knowledge allows a classification of the system as being in its research, development, or deployment stage.

**Figure 1:**
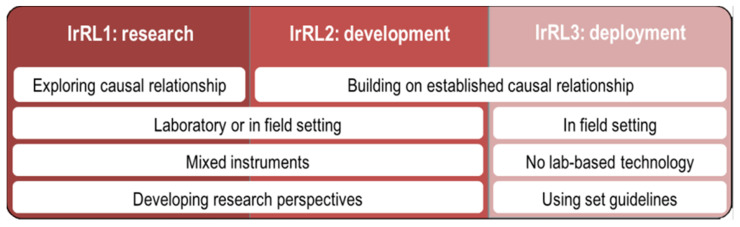
The injury-research readiness level classification model, where different classes of research maturity (IrRL1-3, in columns) are mapped against the following feature domains (rows): knowledge of causal relationship, experimental settings, testing technology, and normative guidelines.

In the context of research on sport-related injuries, we propose that a method is deemed mature for deployment only when it relies on measuring tools that are characterised by high ecological validity (i.e., fully wearable and unobtrusive or markerless), can be applied directly in the field, and is supported by validation studies against an established gold standard or, when validation is not practicable (e.g., tibial peak accelerations), adheres to standardised experimental procedures. Moreover, the approach must be integrated into a testing scenario to justify its validity for preventing, assessing, or informing the recovery from injury. Specifically, the biomechanical quantities used should demonstrate evidence of a causal relationship with the investigated injury, and their interpretation should be driven by specific guidelines (e.g., individual- or population-based normative boundaries, thresholds, or trends). Therefore, we defined and used the following classification (Figure 1):

- IrRL1—research studies not relying on established causal relationships or set guidelines, but rather aiming to find and demonstrate them;
- IrRL2—studies progressing knowledge and building upon demonstrated causal relationships, either in a laboratory or in the field, possibly complementing wearables with lab-based instrumentation;
- IrRL3—studies in which the proposed methods and/or findings are ready for field-based deployment, do not rely on any lab-based or obtrusive technology undermining ecological validity, and use or formalise specific guidelines; the measures indicated as relevant for the study of a specific injury have also been demonstrated to have a causal relationship with the injury itself.

## 3. Results and Discussion

### 3.1. Article Selection and Identification

**Figure 2:**
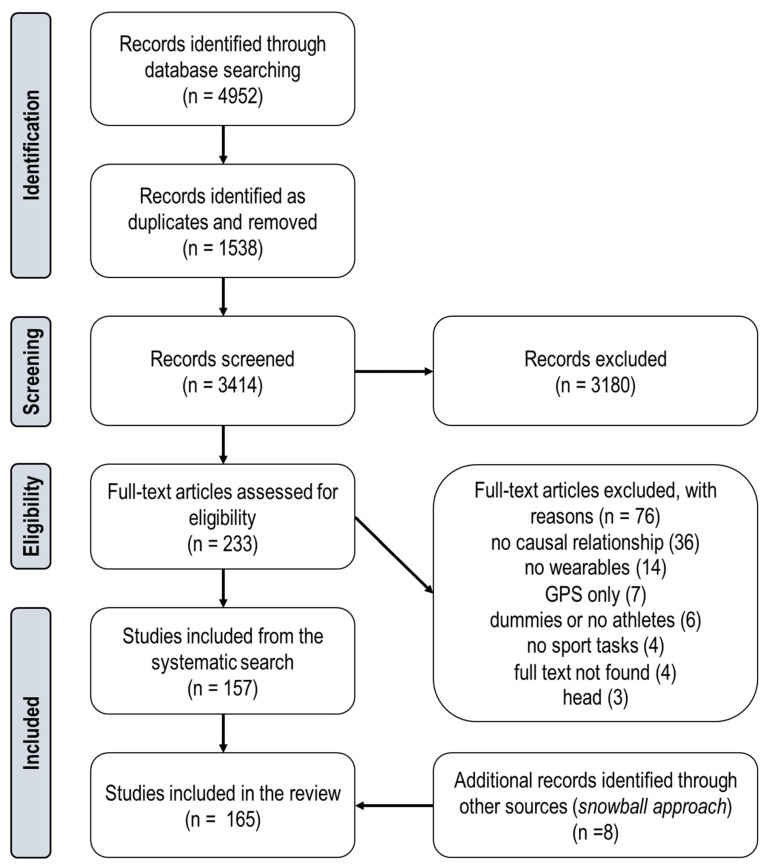
The PRISMA-ScR flowchart, showing the search and selection process.

The screening process (Figure 2) started from 4952 potentially relevant articles, retrieved from the three main databases accessed (Scopus = 2263; Web of Science = 1624; PubMed = 1065). After removing 1538 duplicate items, 3180 publications were excluded based on title and abstract, leaving 234 manuscripts for full-text examination. Discussion in weekly consensus meetings led to a further 77 articles being discarded (most frequent reasons: not including wearables; not including human participants or sport-related activities; and not describing the relationship between biomechanical quantities from wearables and musculoskeletal injury, or not citing references to support that link) and the addition of 8 relevant records from other sources, yielding a total of 165 studies to be considered for the review (Table S2 in the Supplementary Materials) [47,71,72,73,74,75,76,77,78,79,80,81,82,83,84,85,86,87,88,89,90,91,92,93,94,95,96,97,98,99,100,101,102,103,104,105,106,107,108,109,110,111,112,113,114,115,116,117,118,119,120,121,122,123,124,125,126,127,128,129,130,131,132,133,134,135,136,137,138,139,140,141,142,143,144,145,146,147,148,149,150,151,152,153,154,155,156,157,158,159,160,161,162,163,164,165,166,167,168,169,170,171,172,173,174,175,176,177,178,179,180,181,182,183,184,185,186,187,188,189,190,191,192,193,194,195,196,197,198,199,200,201,202,203,204,205,206,207,208,209,210,211,212,213,214,215,216,217,218,219,220,221,222,223,224,225,226,227,228,229,230,231,232,233,234].

Fifty-five percent of the included studies either aimed to establish a relationship between biomechanical quantities and injury (~14%), or included (typically in the introduction) an explanation of this connection by building on evidence from the existing literature (~41%). The remaining 45% did not openly discuss the link between the measures they used and the associated injury, but only mentioned such relationships as possible or hypothetical, citing previous research to justify their choices (Table S2: causal relationship). It therefore appears that nearly half of the studies meeting the primary inclusion criteria and using biomechanical quantities from wearable technologies in the wider context of musculoskeletal sport-related injuries were partly speculative in the construction of their rationale. Indeed, these studies often justified the choice of their measurements by briefly citing the work of other authors, but without discussing the strength of evidence from the previous literature, and rather accepting it even when only hypothetical. Building on robust evidence of the association between specific tests, measures derived thereof, and injury, or trying to establish this relationship, should instead be the starting point for any research in this area, and the basis upon which further evidence is built. This is a crucial element that is not adequately addressed in many of the works scrutinised, and should be better promoted in the design of research programmes and checked during manuscript reviews.

### 3.2. Journals and Years

**Figure 3:**
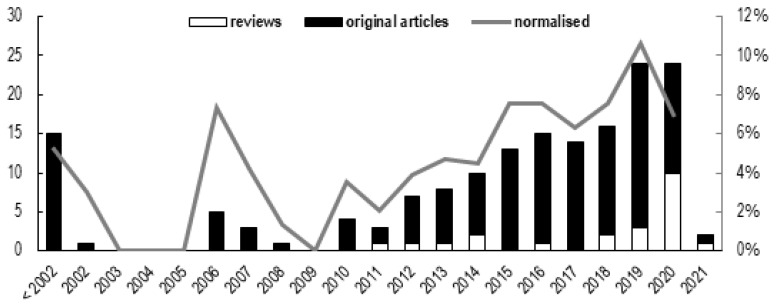
Time distribution of the original studies selected for this scoping review (black), and reviews (white), ordered by publication year. The graph also reports the number of original articles published, normalised (%) to the overall number of manuscripts published in the same year in the broader area of sport injury biomechanics, where this quantity was retrieved using the following MeSH term search in PubMed: ((sport[MeSH Terms]) AND (injury[MeSH Terms])) AND (biomechanics[MeSH Terms])).

The 165 original manuscripts included in the scoping review appeared in 60 different journals, with 11 journals publishing nearly half of the total, and at least 5 relevant articles each (Table S2: journal). The number of articles in the area under scrutiny appears to increase over time (Figure 3), even when publication numbers are normalised to the total of articles published every year in the broader domain of biomechanics of sports injuries. The use of wearables in the area of musculoskeletal sports injuries seems to be increasing in popularity, as also demonstrated by the 22 review papers published between 2011 and January 2021 (Figure 3).

### 3.3. Study Classification and Assessment

#### 3.3.1. Strength of Evidence, Time Setting, and Scope

**Figure 4:**
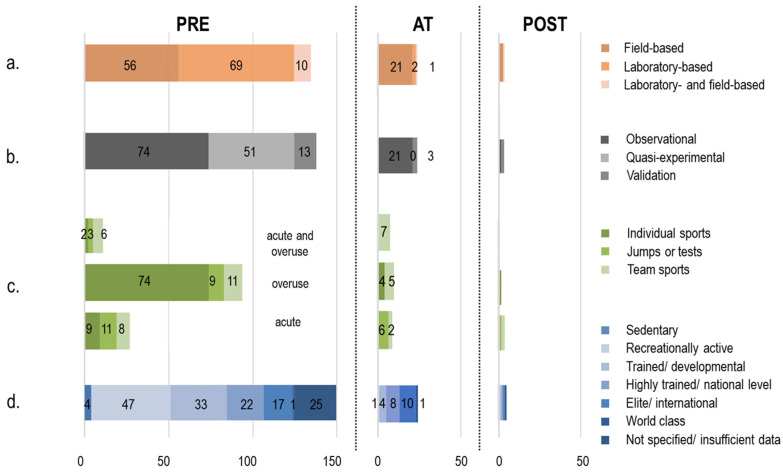
The distribution of studies at different time settings in relation to injury, reported from different perspectives: (**a**) experimental setting; (**b**) strength of evidence; (**c**) injury type; (**d**) level of sport participation. Multiple counts are allowed for injury type and level of sports, since some articles analysed multiple types of injuries and/or tested athletes at different levels of sport.

More than half of the 165 studies scrutinised were observational (96, 58.2%), 51 were quasi-experimental (30.9%), and none could be classified as fully experimental. About 10% (18) attempted validating new methods or protocols against existing standards. Most of the investigations (137, 83%) were at the *pre* stage (44.8% observational, 30.9% quasi-experimental, and 7.3% validation). Considerably fewer (24, 14.5%) attempted to capture actual injuries and could be classified in the *at* group (12.7% observational, 1.8% validation), and only four (2.4%) directly addressed the injury recovery stage and were allocated to the *post* category (0.6% observational, 1.8% validation) (Figure 4 and Table S2: strength of evidence, time setting). The primary focus of the reviewed literature was assessing sport-related injury factors or injury risk in a large majority of studies (124 out of 165), followed by characterising protective devices (15), analysing sport-related injury mechanisms, and gait retraining (11 items each). Few articles addressed post-injury monitoring or return-to-play assessment (4), or attempted to establish injury threshold criteria (2). Two had multiple focuses (Table S2: scope).

Of the 24 retrieved articles classified as *at* (i.e., observing actual injuries as part of the experimental protocol), a majority (54%) focused on workload metrics [75,100,104,126,150,151,160,175,189,197,202,203,234], and often used inertial measurement units (IMUs) to complement information coming from other sources (e.g., global positioning systems, RPE scales). Six articles (25%) adopted a baseline screening approach, whereby biomechanical tests were followed up with injury surveillance over a relatively long period (1 year/season or longer) [47,76,97,143,204]. Only two works—both analysing running—looked into the short-term association between biomechanical quantities and running-related injuries [117] or biomarkers of muscle and kidney injury [232]. Similarly, but only discussing the experimental protocol for a prospective cohort study, one study [118] aimed to use wearables to inform training personalisation and reduce the risk of hamstring injuries in soccer. The only return-to-sport (i.e., *post*) article retrieved [228] that was not a validation study used a single IMU located on the lumbar spine to monitor the recovery from hamstring injury of a single professional soccer player. This case report observed force–velocity interlimb differences, and discussed the sensitivity of the reported metrics in relation to the phase of the competitive season and the occurrence of the injury.

Bringing together the outcomes related to timing with respect to injury, the design of the experiments, and their scope, it appears that currently (a) there is no field-based investigation attempting to detect and capture musculoskeletal injury mechanisms through wearables; (b) there exists little research that exploits wearable technologies to characterise movement behaviours associated with musculoskeletal injuries; and (c) the strength of evidence brought about by the literature is generally not high. As a consequence, a large proportion of studies appear to rely on previous works to discuss injury factors and compare movement behaviours in different groups of participants, but do not directly characterise nor even observe injuries within their experiments. Unfortunately, as outlined in Section 3.1, nearly half of these works—with an even higher prevalence in the *pre* class—did not discuss the validity of the studies they cited to construct their rationale, which in turn affects the validity of their findings and makes them appear more as exploratory investigations of the potential of methods and technologies, rather than as a substantial means to progress knowledge and generate impact on injury factors, mechanisms, or the effectiveness of interventions. For example, amongst the quasi-experimental studies that investigated the effects of interventions, only 29 out of 51 (~56%) (Table S2: causal relationship) clearly introduced the existing evidence of the relationship between the biomechanical quantities chosen as outcome measures and the musculoskeletal injury addressed.

#### 3.3.2. Study Characterisation

##### Experimental Setting

Field-based studies analysing athletes during training, simulated training, or competitions comprised 79 (48%) of the total reviewed, whereas 71 (43%) investigations were performed in laboratory settings; 12 (7%) articles included both settings, and 3 (2%) did not provide enough information to understand where the study was performed (Table S2: setting).

No clear difference was observed in terms of scope between field- and laboratory-based investigations (Table S2: scope), with the majority focusing on the assessment of injury factors (60 (76%) of the field-based and 51 (72%) of the lab-based studies). The remaining laboratory-based studies focused on characterising protective devices (9), primarily in running (5) and basketball (3), gait retraining (8), and understanding running-related injury mechanisms (3). Interestingly, only one of the works dealing with gait retraining was field-based, which may be partly explained by the need to integrate wearable sensors with other equipment (e.g., instrumented treadmill, force plates) and with a pre- vs. post-intervention design relying on ad hoc lab-based sessions. The other field-based studies aimed at understanding sport-related injury mechanisms (8) and characterising protective devices (5) in a large variety of sports disciplines (both individual and team sports), with three studies focusing on post-injury monitoring, and two on setting injury threshold criteria.

When considering the different sport activities (Table S2: sport), 58% of studies performed in laboratory settings focused primarily on running (41), 19 included other individual sports, and only 11 addressed team sport situations. Conversely, field-based investigations considered a wider spectrum of sports, with 43 of them analysing team sports (i.e., soccer, American football, rugby, baseball, Australian football, basketball, handball, and volleyball), 16 on running, and 20 including other individual activities, with a prevalence of winter sports (8 studies out of 11). Studies including both settings primarily focused on running (7 studies out of 15), and the remainder were equally distributed between team and individual sports (4 each).

In 81% (64) of field-based studies, wearable devices were used as the exclusive means to answer the primary research question, or were in any case essential when used in combination with other instrumentation. Conversely, laboratory-based studies were less reliant on wearable technologies, which were classified as essential in only 33 (46%) of them (Table S2: role of IMU-based measurement).

When taking into account the type of injury investigated (Table S2: injury type, tissue affected and body region), in relation to the experimental setting, 79% of laboratory-based studies considered overuse injuries (56), and 83% of them (59) were about lower-limb injuries. Only a few laboratory-based studies focused on upper limbs or spine-related injuries (4 and 5, respectively). Three studies did not provide any information about the type and location of the investigated injury. On the other hand, 39% of field-based investigations addressed acute injuries (31), while 61% looked at overuse injuries (48) and considered a wider variety of injury locations. About half of the articles focused on the lower limbs (38), 14 on the spine, and 9 on the upper limbs. Interestingly, 10 (13%) field-based investigations did not consider a specific body area but, rather, analysed injuries in any body region during rugby, American football, soccer, and volleyball competitions. The remaining eight field-based studies did not specify the type and location of the investigated injury.

##### Injury Type and Location

**Figure 5:**
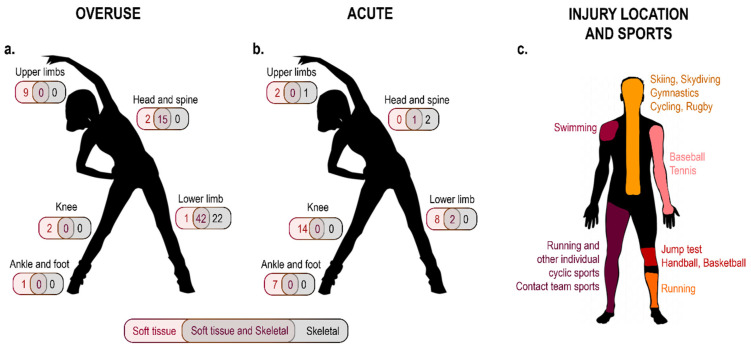
(**a**,**b**) Breakdown of studies (absolute number) by injury location (i.e., ankle and foot, knee, lower limb, head and spine, upper limb), tissue affected (i.e., soft or skeletal), and injury type (acute or overuse). Articles in which the body region was not specified, or considering any injury location, along with articles including both acute and overuse injuries, are not reported in the figure (34 studies in total). (**c**) Body map showing injury location and the associated sports disciplines for which there is prevalence of published research in the areas considered by this review.

The majority of the studies focused on overuse injuries (105, 64%), most of which were located in the lower limbs (65) and the spine (17) (Figure 5a). Acute injuries were addressed in 42 (25%) of the investigations, with a net prevalence of knee (14), ankle (7), and general lower-limb injuries (10) (Figure 5b). The remaining 18 (11%) articles included both acute and overuse injuries primarily related to the lower limbs (6) or any body location (8) (Table S2: injury type and body region).

When considering the type of tissue affected (Table S2: tissue affected), 55 studies (33%) focused on soft-tissue injuries and, among them, 32 were acute, 16 were overuse, and the remaining 7 included both types of injury. Among studies focusing on acute soft-tissue injuries, more than half targeted anterior cruciate ligament tears (12) and ankle sprains (6). Only 25 works out of 165 (15%) considered skeletal injuries, of which a large majority (21) were classified as overuse, and were about stress tibial fractures in running. The rest of the studies (85, 52%) either considered both types of tissues (82) or did not explicitly specify the type of tissue considered (3). Interestingly, about half of the investigations including both soft-tissue and skeletal injuries were on running (38), and used the definition of “running related injury” to refer to them. Running-related injuries were typically described as overuse injuries affecting both soft and skeletal tissues, and located in the lower limbs or, in some cases, the spine.

Acute injuries were investigated primarily in contact team sports (26) or jump/performance tests (12), whereas overuse injuries were observed more during running (63), and in both team and individual sports (42) (e.g., baseball, basketball, volleyball, tennis, cycling, fencing, skating, and skiing). Almost all articles dealing with contact sports (43 out of 48, 90%) focused either on soft-tissue or both soft and skeletal tissue injuries in the lower limbs or at any body region. On the other hand, 96% of studies considering cyclic sports (70 out of 73) analysed overuse leg- (64 dealing with stress tibial fracture) or spine-related (6) injuries. A total of 16 studies out of the 165 reviewed (10%) explicitly dealt with knee injury. All of them considered soft-tissue injuries, and the large majority focused on anterior cruciate ligament tears (12). Only 13 articles focused on upper-limb injuries. Soft-tissue shoulder, elbow, or wrist injuries were considered in studies dealing with baseball (7), tennis (4), and swimming (2). Only one study focused on skeletal injury in the wrist during snowboarding. Finally, vertebral or spine injuries, either in soft or skeletal tissues, were investigated in 22 studies (13%) dealing with different disciplines such as skiing, skydiving, gymnastics, cycling, and rugby (Figure 5c).

##### Population

Ten articles tested multiple types of cohorts (Table S2: participants level of sport, participants number, participants injury history). Over the total of 178 cohorts tested, the majority of articles included in this review analysed recreationally active athletes (28%) and trained/developmental athletes (21%), with highly trained/national level, elite/international, and world-class athletes in 17%, 16%, and 1% of them, respectively. A considerable portion (15%) of works did not specify the level of sport participation.

**Figure 6:**
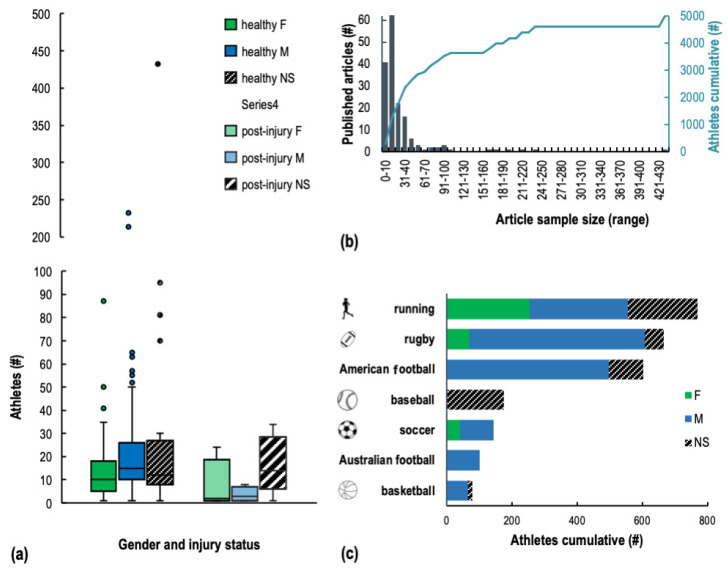
(**a**) Boxplot of healthy and post-injury athletes divided by gender. (**b**) Left: number of athletes analysed in groups of the sample size reported in abscissa; right: their cumulative value. (**c**) Number of athletes, divided by gender, tested in the sports analysed in the 21 studies comprising half of the overall sample of this review (F = females, M = males, NS = gender not specified).

A grand total of 4674 healthy (747 females, 2874 males, 1053 without description of their gender) and 365 injured (46 females, 41 males, 284 without description) athletes were studied across the 165 reviewed works (Figure 6a). A large majority (75%) of the articles studied fewer than 30 participants (median for the grand total: 17 healthy and 8 athletes post-injury), and as few as 22 studies with a sample size from 46 to 432 (median of these 22 studies: 80 athletes) accounted for half of the overall number of participants assessed (Figure 6b). Eight of these 22 investigations tallied up a total of 770 runners of both genders [47,103,124,182,195,209,214,230], whilst 13 studies analysed 1639 male and 70 female players practicing team sports (i.e., basketball [71], American football [104,118,213,234], soccer [185,191], Australian football [160,202], baseball [94,178], and rugby [81,179]) (Figure 6c). These articles on team sports primarily generated the larger prevalence of male athletes in the overall sample analysed in our review. Many articles studying male-dominated team sports did not explicitly specify the gender of their participants; making an implicit assumption that the participants were males would cause an even more unbalanced distribution in the current numbers of male and female populations analysed in the existing literature.

##### Sport and Task

**Figure 7:**
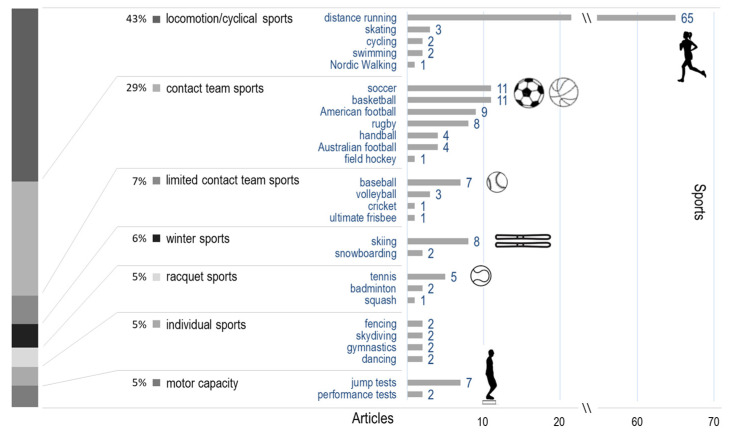
Breakdown of published articles by sport and motor task. Multiple counts are allowed for articles performing comparative studies across sports.

Cyclic and team sports comprised 43% and 36% of the studies analysed, respectively (Figure 7). Winter and racquet sports as well as motor-capacity testing and non-cyclical individual sports all yielded a similar share of interest (5–6%) (Table S2: sport).

The specific motor tasks investigated covered a wide spectrum of gross motor skills (Table 1 and Table S2: motor-task), in many cases with a special focus on tasks more frequently associated with injury events, such as lateral shuffling and sidestep cutting manoeuvres, landing actions with one or two legs, tackling and sustaining physical collisions, overarm throwing, actions simulating injury mechanisms, or sport-specific actions in a variety of disciplines. Articles also focused on actions commonly used in both static (for flexibility, balance, joint mobility, and muscle strength) and dynamic tests.

#### 3.3.3. Technical Features and Validation

Most of the reviewed studies (161, 98%) used inertial sensors, with 104 (63%) employing only 1D or 3D accelerometers, 16 (10%) integrating measures of the accelerometer with those from the gyroscope, and 15 (9%) adding both gyroscope and magnetometer measurements. In three studies (2%), 1D or 3D gyroscopes were used in isolation (Table S2: device type). Nine articles stated that they used IMUs, but did not provide enough information to establish the type of the sensors embedded in the IMU, nor which transducers were used in their investigation. A large majority of articles (75, 45%) did not report any technical information about the technology employed (e.g., mass, dimension, full scale capacity, resolution, buffer, voltage sensitivity; Table S2: device characteristics). In 23 studies (14%), the authors did not even report the sampling frequency at which their sensors were operating (Table S2: device sampling frequency). In the remaining 47 works (28%) describing these specifications, the full-scale capacity of the accelerometers spanned between 6 and 500 g. Thirty (18%) articles reported a full scale for linear accelerations of ≤50 g, whereas most of the gyroscopes reached a maximum of 2000 deg/s for angular velocities.

The most popular commercial systems were Xsens (MVN suit, MTx Sensor, and Awinda; Xsens Technologies B.V., Enschede, The Netherlands) and Catapult (MinimaxX, including all the different versions produced, and OptymEye; Catapult Innovations, Melbourne, Australia), followed by Physilog (Gait Up, Lausanne, CH, Switzerland), Noraxon (Scottsdale, AZ, USA), and Motus Global (Rockville Centre, NY, USA) (Table S2: device brand). In 49 (30%) studies, custom-made or general-purpose devices were adopted, and in 28 (17%) no information was reported about the transducers employed. Little research used pressure (12, 7%) or force (3, 2%) transducers; in some cases (7, 4%), pressure sensors were combined with inertial units. The commercial systems used for pressure and force measurements were mainly produced by Novel (München, Deutschland) and Kistler (Winterthur, Switzerland).

**Figure 8:**
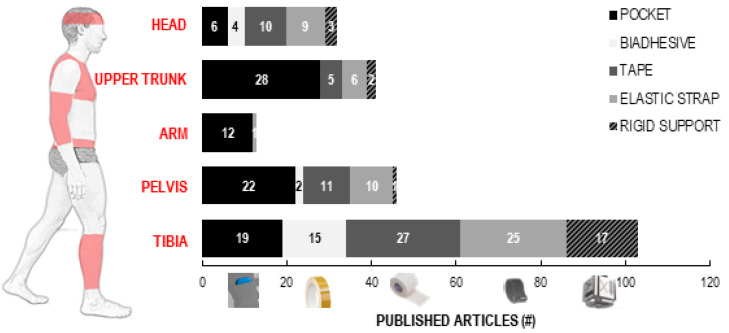
The most used body positions (head, trunk, arm, pelvis, and tibia) and the associated methods of sensor attachment (pocket in a vest/belt/tape, double-side tape directly on the skin, tape above the sensor, elastic strap, and rigid frame). The numbers represent how many studies implemented that type of sensor attachment for each specific location.

In 152 studies (93%), sensors were attached directly to the athlete’s body (Table S2: device number of units): 85 (52%) employed a single-sensor configuration, 53 (32%) used between 2 and 5 sensors, and 10 (6%) utilised more than 5 devices. The tibia was the most popular location (80, 48%), followed by the pelvis (43, 26%) and the trunk (40, 24%) (Table S2: device position). In many articles, the description of body location where the device was placed was too vague to understand the point within the body segment area (e.g., “thigh”, “shank”, “chest”, “upper arm”). Various practices were observed in terms of methods to secure the measuring unit, which also depended on the area of attachment (Table S2: device fixing technique); a lodging pocket was more frequently used for the upper trunk and arm, whereas no preferred fixing technique emerged for tibia and head locations (Figure 8). In 27 studies, the sensors were embedded in or attached to the equipment used (e.g., shoes, tennis racket, boot, ski, bicycle, barbell bar). This was done with glue or tape, or by inserting the transducers into the device, such as for shoe insoles.

Although describing the technical features of the sensor is key to the correct interpretation of data quality and of the meaningfulness of the changes that an intervention may induce, most studies did not report this information in enough detail. As highlighted by recent systematic reviews on inertial sensors for sport performance evaluation [23], and on accelerometry of impact loading in runners [35], reporting the features of the wearable device used—as well as information on the attachment location and fixing methods—is essential.

The sampling frequency should be chosen considering the features of the signal captured and of the noise superimposed. For instance, 99% of the tibial acceleration power during running is below 60 Hz, which led Sheerin et al. [35] to suggest that the sampling frequency of running-related quantities from wearables should be between 300 and 600 Hz. Similarly, it is essential to select the appropriate filtering frequencies, as incorrect filtering can lead to inaccurate interpretation of data [35].

The dimensions, mass, and fixing technique of the sensor should be evaluated in the context of the motor task under analysis and the physical quantities of interest. For sport evaluation in general [23], (1) fixing that restricts the range of movement should be avoided, (2) movement between body segments and sensors should be limited by securing them firmly, (3) the use of elastic belts is not recommended for tasks entailing impacts, and (4) areas close to joints and with soft tissues “wobbling” should be avoided. More specifically, in running [35], (1) a measuring unit with an integrated accelerometer, gyroscope, and magnetometer has higher mass and, thus, the estimation of tibial acceleration is less accurate; (2) measurement error is influenced by the interface between the transducer and the accelerometer-mounting system, where the preload generated by straps or tapes influences the signal (e.g., the tensioning “as much as tolerable” is operator- and participant-dependent). Inserting the sensor in a specially designed pocket of a sport garment is a frequently used solution, which offers an ecologically valid and easily accessible attachment, even in competition settings. However, the use of a pocket may be suboptimal in terms of creating an acceptable attachment of the unit to the participant’s body, and could cause excessive movement artefacts, especially with highly dynamic movements.

Twelve articles in this review contributed to reinforcing the body of methodological guidelines for data acquisition to better capture the content of relevance for risk evaluation. For example, the position and alignment within the body segment, together with the number of measurement axes of the sensor, were found to influence the quality of the investigation for running analysis. (1) Tibial accelerations occur along three directions, and measurements limited to one or two axes may misrepresent the mechanics of the phenomenon [209,235] which, in addition to the calculation of vertical stiffness [144], generally support the use of the resultant acceleration as a quantity less sensitive to alignment problems [209]. (2) Proximal tibial attachments lead to outcomes that are not comparable to those derived from distal locations, due to the influence of the tibial angular velocity and the distance of the device from the ankle [109]. (3) Similarly, shoe-mounted sensors measure higher peak positive accelerations, and are less related to vertical loading rates compared to shank-mounted devices, suggesting that they should not be relied upon if the aim is to monitor modulations of loading rates with changes in running technique (typical of gait retraining) [165]. The comparison of outcomes of known importance for injury research between lab- and field-based conditions highlighted the importance of a research setting specific to the application, suggesting that certain lab constraints may not be appropriate to investigate field-based injuries (e.g., higher intensity of cutting tasks in real games compared to lab-based tests [218]; higher accelerations peaks in field- compared to lab-based running [87]). Findings support measuring tibial impact acceleration in a natural, outdoor environment [124] and, since fatigue may contribute to altering behaviour and variables with time, call for the development of thresholds associated with an increased likelihood of injuries that are specific to field-based conditions [87]. Other specificity concerns have been raised for the interpretation of research results; sex-specific running injuries require sex-specific monitoring to reduce injury risk [47], and differences in sports collisions require sport-specific tackling detection systems [142].

Reporting on the validation of the measurements used, or at least referring to former studies discussing the validity of the approach taken, appears to be relatively widespread (81% of the studies) (Table 2 and Table S2: assessment of measurement quality). However, a considerable portion of the reviewed literature (29 articles, 19%) did not explicitly refer to former validations of the measuring systems and the protocols used within their experiments, or did not assess the absolute and relative repeatability of the measures collected (64, 39%).

#### 3.3.4. Injury-Research Readiness Level

The classification of the articles according to the proposed injury-research readiness level framework identified 75 studies at the *research* level (IrRL1), 70 at the *development* level (IrRL2), and only 20 ready for field-based *deployment* (IrRL3) (Table S2: IrRL). Among the IrRL3 works, few articles (3) fully exploited the potential of biomechanics to detect increased risk of injury (setting injury thresholds [151,213]) or to monitor the effect of interventions (return to play [106]), and none investigated the biomechanics of injury mechanisms.

Of the 20 articles at the *deployment* level (IrRL3) (Table 3), 8 were *pre,* 11 captured real injuries (i.e., classified as *at*), and 1 dealt with return to sport (*post*), forming a time setting distribution that was notably different from that of the overall sample (83%, 14.5%, and 2.4%, respectively). This is consistent with the definition of the *deployment* stage, which requires the ability to perform field-based measures without lab-based technology, to build on established causal relationships between measures and injury mechanisms or risk factors, and to use set guidelines. These requirements are particularly important in the *at* stage, to be able to capture an increase in injury risk at the time of occurrence [75,100,126,143,151,160,197,202,203,213,234], and to guide return to sport *post*-injury based on appropriate biomechanical quantities [106].

Research at IrRL1 is fundamental to identify the cause–consequence relationship between biomechanical factors and injury, which represents the foundations to transition from IrRL1 to IrRL2. Using or establishing guidelines is one of the three key aspects for a study to move from IrRL2 to IrRL3. Within IrRL3, only two articles used or established normative bands or injury threshold criteria. They identified the following quantities as a measure of risk: (1) internal and external training load, provided by accelerometry measures, and perceived wellness, for muscle damage in volleyball players [151]; and (2) symmetry of the region of limb stability (ROLS) for lower-limb injuries of collegiate football players [213]. The potential of these quantities as preventive tools within a surveillance system remains to be assessed. Nine other articles assessing sport-related risk of injury/predictive factors have been building knowledge in this direction, although not providing thresholds:

1. Global navigation satellite systems and accelerometry quantities have been used to obtain workload measures that, despite being widely cited in injury-prevention research, are still considered controversial [23,236,237,238,239]—especially in relation to the ratio between acute and chronic workloads. Daily monitoring of several load-related measures, such as player load—both internal and external—and its variability has been suggested for different sports (i.e., American football [203,234], Australian football [160], baseball [100], soccer [126,197], volleyball [151]), alongside overuse complaints [151] and wellness monitoring to understand the effect of training workloads on injury (American football [75]). Providing individual risk estimates on a daily basis could support practitioners to take better informed decisions while balancing the need to minimize injury risk and maximize athletic performance (Australian football [202]). Similar accelerometry parameters have also been used to describe *whole-body vibrations* to investigate their severity and transmissibility from the skis to the lower back and to the head for different skiing disciplines [207,219].
2. Symmetry reduction has been investigated as a measure of potential risk for lower-limb injuries. Baseline values were analysed in relation to injury history to characterise the region of limb stability, during single-limb stance of basketball players (construct validation [143]).

Many IrRL3 articles contributed to guideline finalisation, providing specific indications of how to assess and use data for monitoring and personalising feedback to prevent injuries or to guide rehabilitation. Running studies at the *deployment* level have typically focused on crucial factors for the assessment of loading capacity and joint stability, including the type of running (treadmill vs. real-word running [124]), running surface [192], and level of fatigue [74]. Baseline values have been obtained for ankle-specific biomechanical measures in runners, in relation to their chronic ankle instability history [157]. Some contributions have focused on developing instruments for personalised feedback with ecological equipment, such as haptic feedback for gait retraining to reduce runners’ tibial acceleration [229], or radar guns to modulate throwing intensity and protect the reconstructed elbow from excess medial torque during rehabilitation [106].

**Figure 9:**
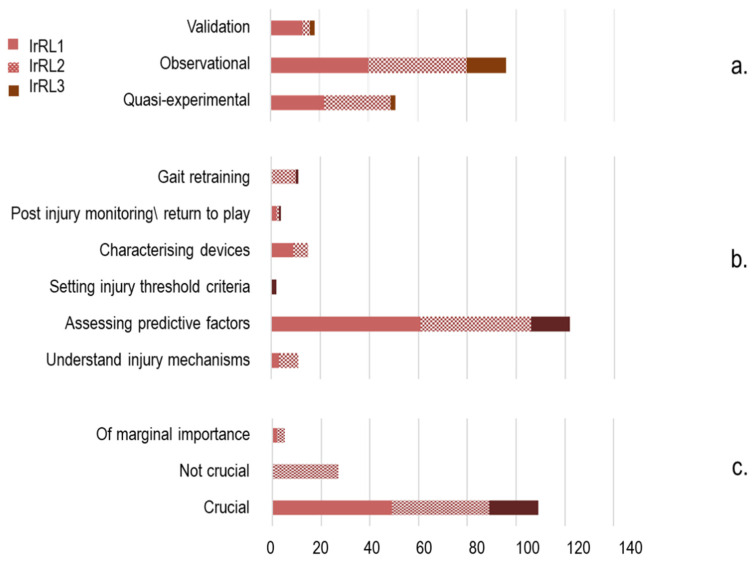
Distribution of studies at different IrRL levels is reported from different perspectives: (**a**) strength of evidence; (**b**) scope; (**c**) relevance of wearable-based parameters.

Zooming out to the three readiness levels (Figure 9), it can be noted that validation studies were mainly classified as being at the *research* level, quasi-experimental and observational studies were more equally distributed between the *research* and *development* levels, and observational studies were the most common design at the *deployment* level (Figure 9a). Studies aiming to understand the biomechanics of injury mechanisms and characterising the role of protective devices were allocated to IrRL1 (3 and 9, respectively) and IrR2 (8 and 6, respectively), whereas the two articles setting injury threshold criteria were at the *deployment* level [151,213] (Figure 9b). All but one article on gait retraining were at IrR2 (10), whereas assessment of sport-related risk of injury/predictive factors was found at all three levels of readiness. The role of wearable-based measures in the different studies ranged from being of marginal importance to being crucial (Figure 9c). This last condition was required for studies to be allocated to the *deployment* level.

**Figure 10:**
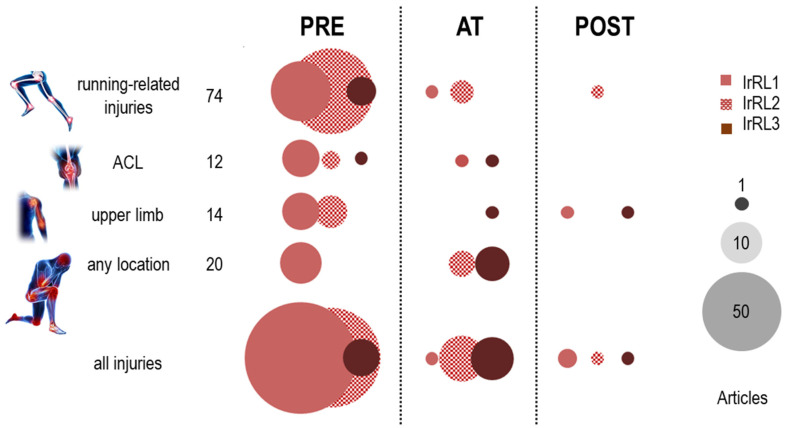
Distribution of studies at different injury-related readiness levels (IrRLs) for different time settings (columns) and injury types (rows).

Looking at injury-related readiness levels from the perspective of specific injuries (Figure 10), we found both *research* and *development* to be similarly represented at the *pre* time setting (72 and 57 articles), and an important share of the *deployment* level articles (8), with a prevalence of studies on running-related injuries [74,124,130,157,192,229]. Research on running-related injuries (74 articles) had nearly double the number of articles at the *development* level compared to the *research* level (44 and 23, respectively). Most were at the *pre* time setting (63), and only four allowed the detection of dangerous changes *at* the time of occurrence and before an injury would eventually occur. The literature on anterior cruciate ligament tears (12) was not extensive, and was predominantly at the *research* level, typically testing different types of jumps and landings to investigate their potential for predicting acute knee injuries; only symmetry of the region of limb stability, at the IrRL3 level, presented a good predictive accuracy [213]. Research on upper-limb injuries (14) focused on baseball- and tennis-related injuries, and included one of the few *deployment* studies at the *post* time setting, with the work focusing on quantitative ecological supervision of throwing rehabilitation of baseball players [106]. A manuscript on measuring acute-to-chronic valgus workload in the upper limbs [100] was also at IrRL3; it appeared to be consistent with seven other articles investigating the effect of workload on any type of injury in team sports. This approach represents the most common *at* scenario, arguably with the highest potential for large-scale *deployment*.

## 4. Conclusions

In this systematic scoping review, we described and discussed the state of the art of biomechanical research using wearable technologies to study musculoskeletal injuries in sport. We aimed to characterise key features of the existing knowledge, identify research trends, and analyse common practices in the design, implementation, and dissemination of experiments. Finally, we proposed a taxonomy to gauge the maturity of sensors and methods in relation to being used in applied settings. This classification framework is a simple yet novel tool that may help drive the efforts of the scientific community towards improving the applied impact of wearables in injury prevention, monitoring, and recovery.

As a potential limitation, the very recent papers published in this area were not included in the temporal range of this scoping review. This was motivated by the very high publication rate that made their inclusion infeasible. As a matter of fact, we can confirm that this potential limitation did not alter the key points raised in the large number of papers included in this review and presented in the Discussion section. The selected articles undoubtedly testify to the widespread interest in the area and an increasing trend in popularity over the last decade.

The analysis led to some key conclusions, which we report, examine as main reflection points, and use to propose some guidelines and good practices for future research and dissemination.

(1) *Articles should explicitly state what the rationale for choosing and analysing specific biomechanical quantities is, and include a justification of what relationship may exist with the injury of interest. When previous literature is cited to support the choice made, the strength of evidence of previous studies should be discussed, together with the context from which that evidence emerged.*

Of the 165 works examined, just over half of them explicitly declared their intention to discover new relationships between biomechanical quantities and injuries, or reported well-formulated arguments built on existing knowledge, to justify the collection and analysis of specific biomechanical quantities. Unfortunately, a sizeable part of the literature failed to include this information, or any critical analysis of the sources supporting their rationale. Many studies simply cited previous research as a justification of their work, even if this literature was only hypothesising—not establishing—links to injury. Failing to build on robust evidence may generate a daisy chain of speculative research, which typically ends in identifying findings or approaches that are of potential interest, yet not demonstrating value for injury-related research. This type of investigation should be called upon to provide a stronger rationale through careful scrutiny during the peer-review process. An in-depth critique of existing knowledge and of the strength of the evidence should instead be the starting point for progressing our biomechanical knowledge and its applied impact for the prevention, characterisation, or management of musculoskeletal injuries.

(2) *More effort should be spent to fully exploit the potential of wearable technologies to detect and characterise injuries when and where they happen, and to monitor and quantify the effects of interventions (preventive or rehabilitative) more regularly.*

Indeed, a small proportion of studies actually aimed to capture musculoskeletal injuries. None attempted to detect or depict injurious events through wearables, with one accidentally capturing an ankle sprain [86], and a single-subject case report describing the process of recovering from a hamstring injury [228]. Most investigations that observed injuries were focused on the quantification of training load, or of acute–chronic workload ratios during training sessions or competition. These metrics, their construction, and their use in different sporting contexts have generated a lively debate in the scientific community [23,236,237,238,239] and should therefore be considered very carefully. Regardless of current concerns as to their use and validity, which go beyond the scope of this review, these load measures only report a summary of the whole-body demands experienced by athletes, and arguably do not fully exploit the potential of wearable technologies to monitor movement behaviour and help identify factors or mechanisms associated with specific injuries of a body location or a tissue. Few studies performed pre-season biomechanical assessment, followed by longer-term injury surveillance, in an attempt to identify movements that may increase injury risk. This experimental design could raise questions about the repeatability of biomechanical quantities over time and, hence, their causal link to injury; at the same time, it could facilitate the early detection of a lack of adaptability to ever-changing task and environment constraints (sports injury forecasting), in association with the concepts and tools used to study the behaviour of self-organising systems [240]. Only two articles—both on running—analysed the relationship between mechanical variables and injury in a shorter timeframe, but were still at an exploratory stage. Finally, there is a large prevalence of observational studies over experimental (no study) and quasi-experimental studies (53 out of 165), which keeps research at a more descriptive level, and does not favour the unveiling of cause–consequence evidence (Figure 4).

(3) *More attention should be paid to selecting an appropriate sample size and type, and to describing it thoroughly.*

Although it is natural to expect that fewer studies on smaller groups are available for elite populations, the disproportion between the number of male and female participants included in the existing literature clearly emerged, and should be carefully considered when designing future experiments. Moreover, studies should systematically report complete information about their population of interest, including gender—which was missing in some articles on male-prevalent disciplines—and level of participation, for which standardised classifications (e.g., [241]) could be adopted, thus simplifying the comparison and summary of outcomes from different works. The great proportion of studies with less than 30 participants is reflective of research designs that do not attempt to capture actual injuries; rather, they typically compare smaller groups of healthy individuals versus athletes who are either prone to recurrent injury or reporting past injury, but considered to be fully recovered at the time of testing. Following up on Conclusion (2), with a view to increasing the effort to collect real injuries or assess the effectiveness of interventions, researchers should more carefully consider the appropriateness of their sample sizes, as opposed to the frequency of the injuries analysed. Since access to participants, resources (e.g., number of sensors, data collection capacity, funding), or both may be complex for biomechanical studies, the opportunity and effectiveness of multicentre studies should be explored and better promoted.

(4) *The quality of the methods, tools, and measures used should be clearly reported, as it is fundamental for the interpretation of the quantities collected. With great power (of the sensors) comes great responsibility (for the researcher)* [242].

A non-negligible part of the studies reviewed did not carry out nor report on existing validations of their measuring equipment, protocols, or settings. Two works included information about former validation studies, but without making sure that these tests had been performed on sensors of the same type, making them clearly inappropriate. Discussing aspects such as accuracy, reliability, existing guidelines, and the possible influence of experimental choices on outcome measures (e.g., physical characteristics of the measuring unit; settings such as frequency, resolution, sensitivity, and full-scale capacity; exact attachment location of the sensors; fixing methods, as far as sensors are concerned, but also running surface, fatigue, and measurement protocols) is an essential yet often overlooked element, and should be better taken care of. Indeed, it impacts the interpretation of results, the ability to replicate a study, and the possibility to compare results from different articles. For example, in some cases, we even found it difficult to understand what sensor or element of an integrated system (e.g., in magneto-inertial units with embedded global navigation satellite systems) was used to extract the outcome metrics selected. Finally, results obtained under controlled lab conditions should be handled with care; for example, field-based demands such as fatigue or high-intensity drills may prove difficult to replicate in a laboratory, and it is impossible to investigate acute injuries with lab-based testing. Conversely, guidance and thresholds identified in a lab may be difficult to transfer to field-based situations.

The continuous progress in wearable sensors offers many opportunities to collect data on many athletes simultaneously, non-obtrusively, for long periods, and in field-based situations. However, the great “power” that even consumer-level technologies (e.g., smart phones, watches, pods) currently offer does not come free of problems, such as those associated with the management and processing of large datasets affected by noise. Improvement in data quality and the ability to extract meaningful information from large databases affected by noise are issues that researchers need to carefully consider and address. The advances in data science techniques (e.g., machine learning approaches) could play a great role in this perspective; however, an appropriate and effective use of wearable technologies should be informed by a thorough awareness of sensor limitations, in terms of both measurement quality and range of suitable applications. The following list includes some of the most critical issues, but the reader is referred to the recent recommendations in [23,35,43,243] for more complete guidance on countermeasures:

- Noise can be assumed to be proportional to task dynamics, which suggests reducing the mass and dimensions of the devices to the minimum possible;
- Appropriate full-scale ranges should be selected according to the measure of interest [244,245];
- The estimation of displacement (linear and angular) in magneto-inertial measurement units (MIMUs) is influenced by drift caused by the combination of MEMS physical properties and finite integration [246], which should be spot-checked (e.g., [247,248]) and requires compensation through ad hoc algorithms [247,249];
- Errors in orientation estimation can originate from ferromagnetic disturbances when the magnetometer is exploited—particularly indoors [250,251];
- Soft-tissue artefacts and fixation techniques affect the validity of measurements, as they can reduce or accentuate the real movement of the body segments that the sensors are attached to [252,253,254]. Soft-tissue artefacts are particularly problematic and complex to deal with, as they are strongly sensitive to task [235], participant [255], site and method of unit attachment [256,257], and unit removal and replacement [258], calling for recommendations of sensor types and placements specific to sports and parameters of interest (e.g., [259]);
- Bespoke calibration techniques may be required to guarantee enough accuracy and reliability throughout the movement of interest and the environmental conditions under which it is performed [260];
- Anatomical calibration should be carefully performed to ensure interpretation and comparison within and between athletes, at least through alignment with gravity during a neutral standing posture, or alternatively functional or point-based—especially when assessing joint kinematics [261,262];
- Practically, splitting data collection into short trials of less than 30 s, collecting as many trials as possible per condition tested, and performing a pose calibration for each of those conditions typically leads to better measures [79].

(5) *The road towards applied impact offers many opportunities… but is long and difficult, take “the one less travelled by, […]” and it will make “all the difference”* [263]!

The injury-related readiness level classification system we outlined and applied to the selected article database showed that only few investigations are at a stage of maturity where their findings could be deployed for field-based use. This taxonomy allows the identification of applications, sports, and bodily tissues/areas for which more biomechanical research would be beneficial. Indeed, the highest class of readiness relies on the findings from the lower levels of maturity, and mapping the extent of work and knowledge available at the different stages will help to direct research endeavours and appreciate the distance from final knowledge transfer. Sport biomechanics can offer a fundamental contribution to the prevention, assessment, and recovery from musculoskeletal injury and, through the use of wearables, can also play a key role in multiple elements of the many models of injury prevention [3,8,9] and injury causation [10,11,12] that have been proposed in the literature. For example, it can help identify the factors predisposing an athlete to injury or the events inciting an injury. It can describe and quantify the mechanisms of an injury and the movement behaviours associated with it. It could contribute to the creation of decision-support systems based on quantitative analysis, and help with monitoring interventions and the compliance of athletes and practitioners to those interventions. Our readiness analysis has shown that this last area is particularly lacking.

Performing real-time predictions based on machine learning and providing real-time feedback has a huge potential to both enhance the athlete’s performance, through movement recognition [264] and technique correction [23], and to assess injury predictive factors [265] and evaluate them within long-term monitoring of injury-forecasting systems [240]. This is particularly relevant for athletes with disabilities, where personalised monitoring systems can be developed to anticipate injuries specific to their disability [26]. It also challenges the research community to develop more intelligent, real-time, accurate information, making it user-friendly and offering coaches and athletes actionable insights based on context-specific evaluation frameworks and on the ability to identify correct forms and common deviations of specific movements according to an agreed-upon clinical consensus [266]. Indeed, personalised and effective wearable technology should be rooted in a thorough understanding of the user’s experience, attitudes, and opinions which, if not properly considered, can severely hamper the potential of applications [267].

To enable and maximise the contribution of wearable technology and biomechanics, more coordinated efforts should be spent so that a systematic and sound progression from basic research to knowledge transfer is generated. Discovering biomechanical relationships with injuries and their mechanisms is difficult, and often requires prospective analysis of large cohorts of athletes; this is typically costly, lengthy, and overall difficult to implement for a single research group. Multicentre studies would most likely facilitate this process. That being said, the ever-increasing availability of wearable technologies and smart textiles [268] able to measure a large spectrum of biomechanical and physiological quantities would conceivably boost the research in the abovementioned desirable direction. The integration of multiple sensors of different types, together with a systemic rather than a local approach when studying injury in sports, represents two crucial elements that deserve attention for future research.

## Figures and Tables

**Table 1:** Breakdown of motor tasks investigated, classified by different gross motor skills or aims of the motor task.

Locomotion	Agility runs (lateral shuffling and sidestep cutting manoeuvres); Bicycling (road climb and single-track climb, downhill); Jumping (vertical drop jump, landing with one or two legs, countermovement jump, standing long jump, double-legged hops, rebound jumps, single-leg drift jump); Skating (short-track speed, treadmill inline); Running (overground, trail and treadmill, level/uphill/downhill, outdoor distance running, jogging, Nordic walking, walking); Skiing and snowboarding (alpine skiing, ski jumping, carved turns); Gymnastics routines (back walkover and back handsprings); Swimming (freestyle); Sport-specific actions: rugby, soccer matches and training sessions, volleyball, baseball, basketball, American and Australian football tasks, flamenco, ballet dance steps
Accelerating or decelerating masses	Tackling and sustaining physical collisions (scrummaging, shoulder charge tackles, strikes, hit-ups and impacts/ collisions)
Throwing or hitting	Overarm movements (tennis serve/one-hand or backhand drive, pitching, cricket bowling, baseball and volleyball throws and serves); Sidearm movements (fencing lunge)
Simulating injury mechanisms	Ankle sprain motion; Falls
Testing	Static tests for flexibility, balance, joint mobility, and muscle strength (single-limb stance, modified Star Excursion Balance Test, lunge, sit-and-reach, adductor squeeze, planar and multiplanar single-leg hopping); Dynamic tests (shuttle run, sidestep, change of direction and acceleration, agility T-Test, drills designed to reflect the mechanism of ankle sprain injury)

**Table 2:** Breakdown of approaches used for sensor validation.

Perform sensors’ validation within the cited article (22)	- Compared to gold standards (e.g., stereophotogrammetry, force platforms, high-speed video, or photocells) (16); - Comparing classification results against human\validated software classification (6).
Perform pilot studies (8)	- Improving fixing or accuracy;
Refer to former validation studies (42)	- Referring to accuracy and/or reliability of the same sensors as obtained in other validation studies.
Refer to ad hoc procedures for the performed measures (64)	- Describing procedures for tibial acceleration or shock propagation measures (48); - Referring to procedures other than tibial shock in the literature (6); - Referring to international ISO standards (3); - Referring to vendors’ procedures (6).

**Table 3:** Studies at the *deployment* level (IrRL3), divided by time setting and scope. Icons represent the body region related to the injury and the sport involved.

PRE			AT		POST
assessing sport-related injury factors or injury risk					
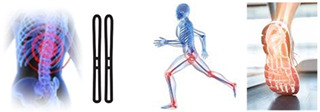			* 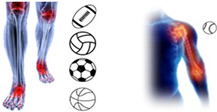 *		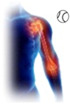
[207,219]	[74,124]	[157,192]	[75,126,143,151,160,197,202,203,234]	[100]	[106]
gait retraining			establishing injury threshold criteria		post injury monitoring
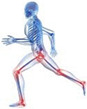 [229]			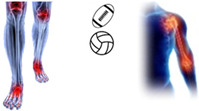 [151,213]		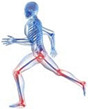 [130]

## Supplementary Materials

The following supporting information can be downloaded at: https://www.mdpi.com/article/10.3390/s22093225/s1, a database with the details of all articles that are not reviews and were included in the systematic review.
