# Supplementary material for: The Use of Wearable Sensors for Preventing, Assessing, and Informing Recovery from Sport-Related Musculoskeletal Injuries: A Systematic Scoping Review
Source: Sensors (Basel). 2022 Apr 22;22(9):3225. doi: 10.3390/s22093225 (PMC9105988; doi:10.3390/s22093225)
Supplement: Supplementary file 1 [file sensors-22-03225-s001.zip › Table S1.pdf]

sensors

MDPI

Supplementary Materials

Table S1. Boolean search strategy.

Database keywords
**Web of Science:** *(accelerometry OR accelerometer OR gyroscope OR inertial sensor OR inertial measurement unit OR wearable sensor OR wearable system OR wearable device OR IMU* OR MEMS OR (force AND wearable) OR (pressure sensor AND wearable))*<br>*AND (Sport OR Baseball OR Basketball OR Bicycling OR Boxing OR Football OR Golf OR Gymnastics OR Hockey OR Martial Arts OR Tai Ji OR Karate OR Taekwondo OR Mountaineering OR Racquet Sports OR Tennis OR Cricket OR Softball OR Badminton OR Running OR Rugby OR Jogging OR Skating OR Snow Sports OR Ski* OR Soccer OR Snowboard OR Swimming OR Diving OR Track and Field OR Volleyball OR Weight Lifting OR Wrestling OR surface OR equipment OR jump* OR land* OR lacrosse OR handball)*<br>*AND (injur* OR risk* OR impact* OR prevent* OR shock* OR load*)*<br>*AND (Humans OR Athletes OR collegiate OR junior OR elite)*<br>*NOT (Patients OR animals OR physical activity OR energy expenditure)*<br>*Limit to English*
**Scopus:** ("accelerometry" OR "accelerometer" OR "gyroscope" OR "inertial sensor" OR "inertial measurement unit*" OR "wearable sensor" OR "wearable system" OR "wearable device" OR "IMU*" OR "MEMS" OR ("force" AND "wearable") OR ("pressure sensor" AND "wearable"))<br>AND ("sport" OR "Baseball" OR "Basketball" OR "bicycling" OR "Boxing" OR "Football" OR "Golf" OR "Gymnastics" OR "Hockey" OR "Martial Arts" OR "Tai Ji" OR "Karate" OR "Taekwondo" OR "Mountaineering" OR "Racquet Sports" OR "Tennis" OR "Cricket" OR "Softball" OR "Badminton" OR "Running" OR "Jogging" OR "rugby" OR "skating" OR "Snow Sports" OR "ski*" OR "Soccer" OR "Snowboard" OR "Swimming" OR "Diving" OR "Track and Field" OR "Volleyball" OR "Weight Lifting" OR "Wrestling" OR "surface" OR "equipment" OR "jump*" OR "land*" OR "lacrosse" OR "handball")<br>AND (humans OR athletes OR collegiate OR junior OR elite)<br>AND ("sports injur*" OR "strain injur*" OR "knee injur*" OR "stress injur*" OR "overuse injur*" OR "athletic injur*" OR "anterior cruciate ligament injur*" OR "lower limb injur*" OR "upper limb injur*" OR "injury risk*" OR "knee impact*" OR "foot impact*" OR "shoulder impact*" OR "impact shock*" OR "tibial shock*" OR "injury prevention" OR "shock attenuation" OR "impact load*" OR "accelerometer load*" OR "physical collision*" OR "running load*" OR "tibial load*" OR "inertial load*" OR ("low back" AND "injury"))<br>AND NOT (patients OR animals OR "physical activity" OR "energy expenditure") AND (LIMIT-TO (DOCTYPE, "ar") OR LIMIT-TO (DOCTYPE, "re") OR LIMIT-TO (DOCTYPE, "ip")) AND (LIMIT-TO (LANGUAGE, "English"))
**PubMed:** ("accelerometry" OR "accelerometer" OR "gyroscope" OR "inertial sensor" OR "inertial measurement unit" OR "wearable sensor" OR "wearable system" OR "wearable device" OR "IMU" OR "IMUs" OR "MEMS" OR (force AND wearable) OR ("pressure sensor" AND wearable))<br>AND (Sports[Mesh] Baseball OR Basketball OR Bicycling OR Boxing OR Football OR Golf OR Gymnastics OR Hockey OR Martial Arts OR Tai Ji OR Karate OR Taekwondo OR Mountaineering OR Racquet Sports OR Tennis OR Cricket OR Softball OR Badminton OR Running OR Rugby OR Jogging OR Skating OR Snow Sports OR Ski OR Soccer OR Snowboard OR Swimming OR Diving OR Track and Field OR Volleyball OR Weight Lifting OR Wrestling OR surface OR equipment OR land OR landing OR jump* OR lacrosse OR handball)<br>AND ("injur*" OR "risk*" OR "impact*" OR "prevent*" OR "shock*" OR "load*")<br>AND (Humans[Mesh] OR Athletes[Mesh] OR collegiate OR junior OR elite)<br>NOT (patients OR animals OR "physical activity" OR "energy expenditure")<br>AND ("english"[Language])
